# Air-liquid interface exposure of A549 human lung cells to characterize the hazard potential of a gaseous bio-hybrid fuel blend

**DOI:** 10.1371/journal.pone.0300772

**Published:** 2024-06-24

**Authors:** Jonas Daniel, Ariel A. Schönberger Alvarez, Pia te Heesen, Bastian Lehrheuer, Stefan Pischinger, Henner Hollert, Martina Roß-Nickoll, Miaomiao Du

**Affiliations:** 1 Institute for Environmental Research, RWTH Aachen University, Aachen, Germany; 2 TME—Chair of Thermodynamics of Mobile Energy Conversion Systems, RWTH Aachen University, Aachen, Germany; 3 Department Evolutionary Ecology & Environmental Toxicology (E3T), Faculty Biological Sciences (FB15), Goethe University Frankfurt, Frankfurt, Germany; 4 Department Environmental Media Related Ecotoxicology, Fraunhofer Institute for Molecular Biology and Applied Ecology IME, Schmallenberg, Germany; Chiang Mai University, THAILAND

## Abstract

Gaseous and semi-volatile organic compounds emitted by the transport sector contribute to air pollution and have adverse effects on human health. To reduce harmful effects to the environment as well as to humans, renewable and sustainable bio-hybrid fuels are explored and investigated in the cluster of excellence “The Fuel Science Center” at RWTH Aachen University. However, data on the effects of bio-hybrid fuels on human health is scarce, leaving a data gap regarding their hazard potential. To help close this data gap, this study investigates potential toxic effects of a Ketone-Ester-Alcohol-Alkane (KEAA) fuel blend on A549 human lung cells. Experiments were performed using a commercially available air-liquid interface exposure system which was optimized beforehand. Then, cells were exposed at the air-liquid interface to 50–2000 ppm C_3.7_ of gaseous KEAA for 1 h. After a 24 h recovery period in the incubator, cells treated with 500 ppm C_3.7_ KEAA showed significant lower metabolic activity and cells treated with 50, 250, 500 and 1000 ppm C_3.7_ KEAA showed significant higher cytotoxicity compared to controls. Our data support the international occupational exposure limits of the single KEAA constituents. This finding applies only to the exposure scenario tested in this study and is difficult to extrapolate to the complex *in vivo* situation.

## 1. Introduction

Gaseous and semi volatile organic compounds emitted by the transport sector contribute to air pollution and are proven to have adverse effects on human health [1–3]. The WHO estimated that 4.2 million premature deaths worldwide were caused by ambient outdoor air pollution in 2019 [4], mainly due to cardiovascular and respiratory diseases, and cancers. Addressing this problem, one target of the UN’s Sustainable Development Goals is to substantially reduce the number of deaths and illnesses from hazardous chemicals and air contamination (target no. 3.9) [5]. To reduce emissions and harmful effects to the environment as well as to humans, renewable and sustainable bio-based alternatives to fossil fuels are explored and investigated globally [6–8]. Recent studies by Landwehr et al. focus on the effects of exhaust aerosol produced from the combustion of different bio-based fuels in an engine [9]. When combusted, the exhaust of bio-based containing fuels, i.e., biodiesel was shown to be less toxic to human lung cells compared to the exhaust of fossil fuels [10]. Depending on the feedstock biodiesel is made from the toxicity to human lung cells varies [11]. However, while it is clear that emissions after the combustion of fuels do pose a risk to human health, little attention is paid to effects of fuel emissions before combustion, i.e., the vapor of unburnt fuel itself. For example, both a significant decrease in cell viability and a significant increase in pro-inflammatory response were observed in human lung cells exposed to unburnt volatile gasoline constituents [12, 13]. Hazards from fuel vapor may arise from occupational inhalation exposure during transportation and storage of fuels [14, 15]. Relevant exposure scenarios include long-term exposure of workers at gas stations and to its residents [16, 17]. For example, the nasal mucociliary transport time of gas station workers was positively correlated with the length of work [18]. An impaired mucociliary clearance, a self-clearing mechanism, might not function well to remove pathogens from the respiratory tract, leading to infections of the airways [19]. Also, exposure to fuel vapor may play a role in bacterial colonization of the respiratory tract in fuel workers. For example, AlWakeel found bacteria with over 50% resistance to first-line antibiotics, e.g., ampicillin, which might complicate curing respiratory infections [20]. Further, vehicle mechanics performing gasoline-related tasks such as draining a gas tank or changing a fuel pump are directly exposed to high short-term airborne concentrations of fuel vapor exceeding occupational exposure limits (OELs) [21].

The OELs state the maximum allowed airborne concentration of a single chemical during a work shift [22]. However, naturally fuels comprise of several different constituents and may be further blended with additives to improve their stability and to promote cleaner combustion [23]. Therefore, it is important to investigate potential health effects of fuel blend vapors, which can be difficult to predict depending on the number of single components [24]. One promising bio-based fuel blend candidate, a Ketone-Ester-Alcohol-Alkane blend (KEAA), is currently being investigated in the cluster of excellence “The Fuel Science Center” (FSC) at RWTH Aachen University. KEAA can be made via a bio-hybrid production route, which uses a combination of biomass, CO_2_, H_2_ and renewable energy as building blocks [25, 26]. Data on effects of so-called bio-hybrid fuel blends on human health is scarce, leaving a data gap regarding their hazard potential. For example, moderate ecotoxicity of KEAA was shown by Ackermann et al. [27]. According to the principle of Green Toxicology [28], a characterization of this hazard potential should be conducted in parallel to the development of and prior to manufacturing and distribution of bio-hybrid fuels itself.

To help close this data gap, this study investigates potential toxic effects of KEAA fuel vapor on human lung cells, using the well-characterized human lung cell line A549 [29, 30] as an *in vitro* lung model. To increase both sensitivity of the lung model as well as relevance of the toxicological data, a state-of-the-art air-liquid interface (ALI) continuous flow exposure system is used [31, 32]. In the exposure system a direct interaction between cells at ALI conditions and fuel vapor takes place, representing the physiology and exposure of human lung cells more accurately compared to cells submerged in a culture medium [33–35]. Therefore, ALI exposure has become more popular in the last few years [36] since it is also a promising alternative to *in vivo* animal experiments, promoting the 3R principle (replacement, refinement, reduction) [31]. Achieving reliability, however, ALI exposure requires a complex technical setup and monitoring of important parameters [37–39], e.g., temperature and relative humidity, which is also highlighted in the present study.

The aim of this study was to establish an *in vitro* ALI exposure system to investigate the potential toxicity of a bio-hybrid fuel blend vapor on A549 human lung cells. A promising Ketone-Ester-Alcohol-Alkane (KEAA) gasoline-like fuel blend candidate was chosen based on previous work [26, 27]. The approach presented here includes the generation and chemical analysis of the test gas as well as the adjustment and application of a commercially available ALI exposure system for ALI experiments.

## 2. Materials and methods

### 2.1 Fuel blend

The KEAA fuel blend [27] used in the present study consists of 41 mol% 3-methylbutanone, 25 mol% ethanol, 16 mol% methyl acetate, 12 mol% ethyl acetate, 4 mol% pentane and 2 mol% methanol. All constituents (analytical grade) were purchased from VWR International GmbH and were blended in the lab.

### 2.2 Generation and analysis of the feed gas

**Fig 1:**
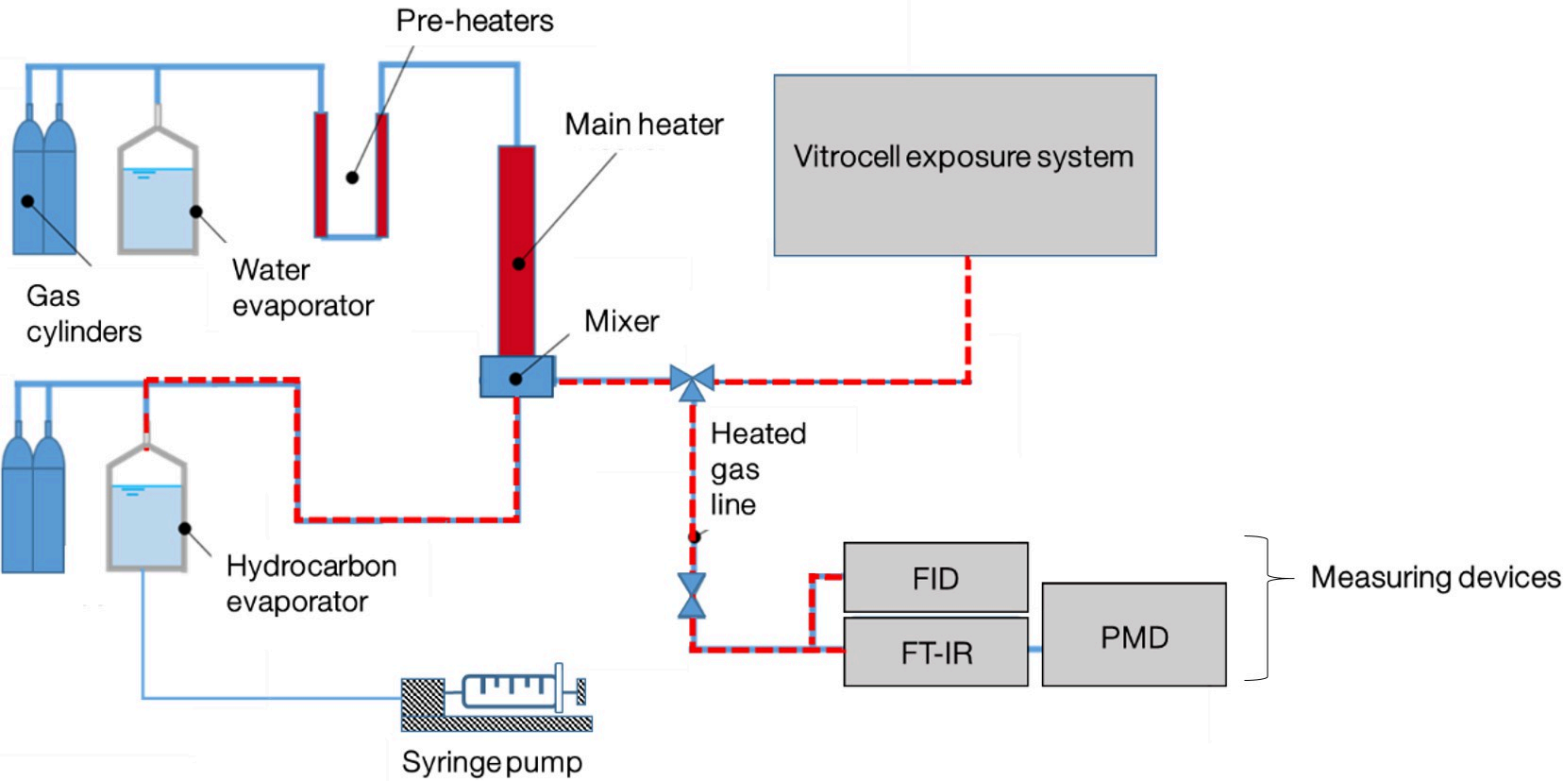
Schematic setup of the model gas test bench with the connected Vitrocell exposure system. The liquid hydrocarbons (HCs) were dosed into an evaporator using a syringe pump. Evaporated HCs were transported via a humidified gas stream (N_2_:O_2_, 80:20, % v/v) through heated pipes to the Vitrocell exposure system and measuring devices. HC species were measured using a Fourier-transform infrared (FT-IR) analyzer and for total HC amount a flame-ionization detector (FID) was used. For O_2_ measurement a paramagnetic detector (PMD) was used.

The feed gas for all ALI experiments was generated on a custom-built model gas test bench (MGTB) made from grade 2 titanium (Fig 1). The feed gas is convectively heated with a closed loop control to achieve the desired temperature. All gas pipes are as well made from titanium and heated to 37°C to prevent condensation of components from the feed gas. Various mass flow controllers (MFC) type SLA5850 (Brooks Instrument LLC, Hatfield, PA, USA) were used to regulate the flows of N_2_ (99.999 vol.%, Nippon Gas), O_2_ (99.999 vol.%, Westfalen Gas) and NO_2_ (5000 ppm in N_2_, Westfalen Gas). A HovaPOR LF-1200 (IAS GmbH, Oberursel, Germany) was used to supply water, vaporized in another part of the N_2_ flow, to humidify the feed gas. The liquid KEAA hydrocarbons were dosed with a syringe pump (Cetoni GmbH, Korbußen, Germany) and evaporated in a self-designed titanium evaporator which also uses a part of the N_2_ flow. Verstraelen et al. used a comparable setup to evaporate liquid ethylbenzene to expose A549 human lung cells to its gaseous form [12]. Liu et al. evaporated a BTEX mixture to expose A549 human lung cells [13]. A variety of different measuring devices was used for the gas analysis bypassing the Vitrocell exposure system: A MultiGas 2030 HS Fourier-transform infrared (FT-IR) spectroscopy gas analyzer (MKS Instruments Inc., Andover, MA, USA) for measuring hydrocarbon species (HC) and a flame-ionization detector (FID) (Thermo-FID MP by SK-Elektronik GmbH, Leverkusen, Germany) for total HC amount normalized to C_3_ and for NO_2_ was used; in serial arrangement an H_2_O condenser for measuring water content and a measurement system (FEV Europe GmbH, Aachen, Germany) containing a paramagnetic detector (PMD) for O_2_ measurement was installed. The gas matrix used was adapted to the different experiments, using the same total gas flow of 17.4 L/min (standard conditions/NIST) with a N_2_ gas flow as equilibrium.

### 2.3 Dilution of the feed gas

From the total gas flow of the MGTB, a 5 L/min feed gas stream was extracted and transported to the Vitrocell exposure system. In the exposure system, the feed gas stream was diluted 1:5 with humidified clean air in both dilutors, which yields a 1:5 dilution (first dilutor) and a 1:25 dilution (second dilutor), respectively. Therefore, two concentrations of the KEAA blend were tested in parallel per experiment. In total, KEAA concentrations ranging from 50–2000 ppm C_3.7_ were tested. Concentrations of the single KEAA constituents after dilution were calculated from measured concentrations in the undiluted feed gas and are presented in Table 1.

**Table 1:** Calculated concentrations of single KEAA constituents after dilution based on measurements of the undiluted feed gas.

	Total HC concentration C_3.7_ ppm							
	50	100	200	250	400	500	1000	2000
Substance	ppm							
3-Methylbutanone	15.9	31.8	63.6	79.5	127.1	158.9	317.8	635.6
Ethanol	9.9	19.8	39.7	49.6	79.3	99.2	198.3	396.6
Methyl acetate	6.1	12.3	24.5	30.7	49.0	61.3	122.6	245.2
Ethyl acetate	4.9	9.8	19.7	24.6	39.4	49.2	98.4	196.8
Pentane	1.6	3.1	6.3	7.9	12.6	15.7	31.4	62.8
Methanol	0.9	1.8	3.5	4.4	7.1	8.9	17.7	35.4

**Fig 2:**
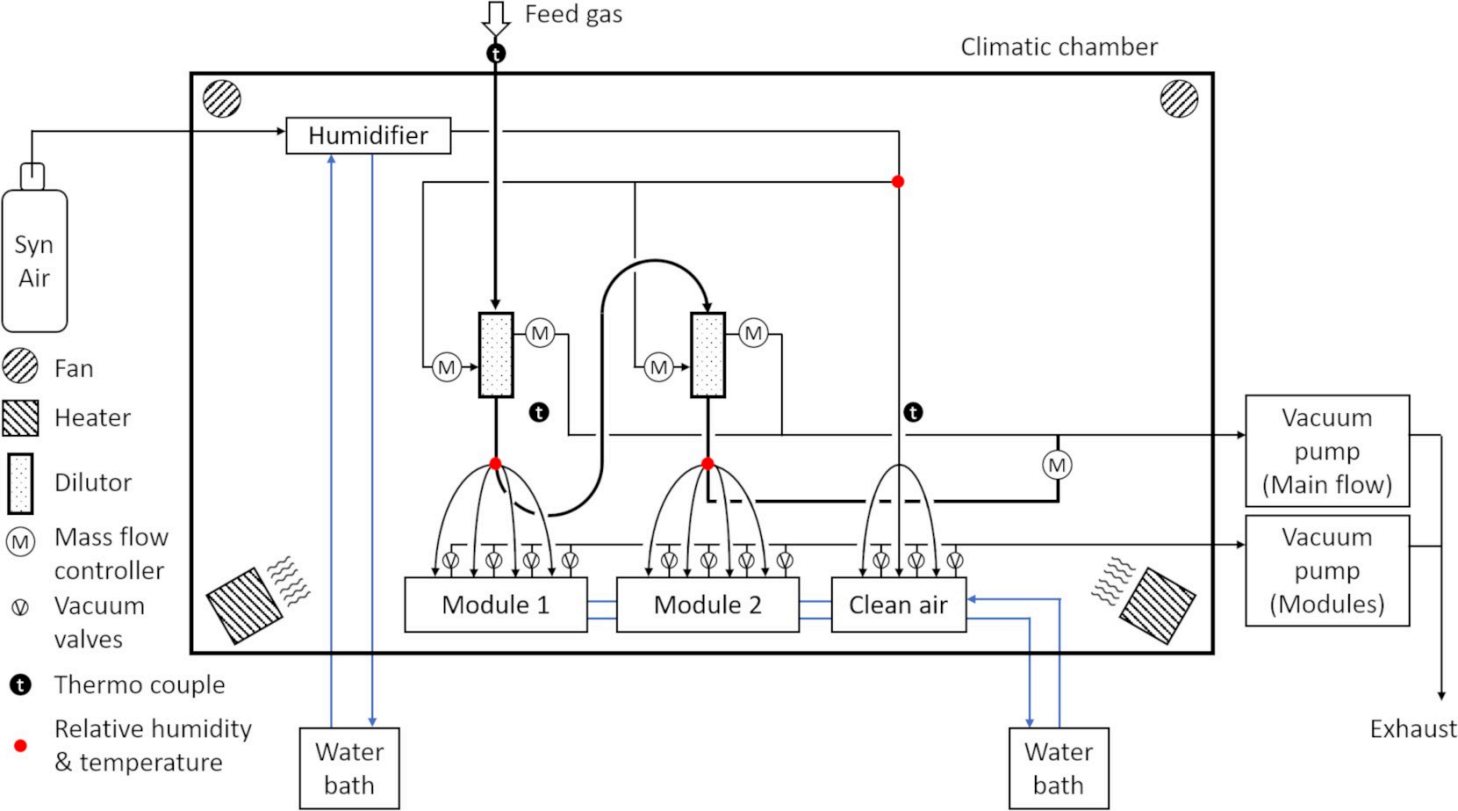
Schematic of the exposure system used to expose cells at air-liquid interface to gaseous chemicals. (courtesy of Vitrocell Systems GmbH).

Humidified clean air was generated from synthetic air (grade 5.0) passing a humidifier (Naphion cartridge) connected to a water bath (KISS 104A, Peter Huber Kältemaschinenbau AG, Offenburg, Germany) (Fig 2). Temperature and relative humidity of humidified clean air (37°C, 85% rH) were measured upstream of the dilutors using a RH/T sensor (testo 645, testo SE & Co. KGaA, Lenzkirch, Germany).

NO_2_ spiked feed gas from the MGTB was diluted 1:5 with humidified clean air in one dilutor of the exposure system to a nominal concentration of 10 ppm.

### 2.4 Air-liquid interface (ALI) exposure system

The exposure system used for ALI experiments was bought from Vitrocell Systems GmbH (Waldkirch, Germany). A schematic setup of the exposure system is shown in Fig 2. The whole system includes several devices for controlling mass flow, dilution, humidity, and temperature of the test gas. First, feed gas enters the main flow pipe (stainless steel) of the exposure system by applying negative pressure using a vacuum pump (SECO SV 1008 C, Busch Vacuum Solutions, Maulburg, Germany). Then, the feed gas passes two dilutors in series (total dilution ratio up to 1:100), in which parts of the feed gas are extracted and humidified clean air is added using mass flow controllers (MFC Serie 358, ANALYT-MTC Messtechnik GmbH, Müllheim, Germany). After each dilutor, the test gas is extracted from the main flow via four isokinetic sampling probes, each directly connected to one position of a VITROCELL 6/4 stainless steel exposure module (Module 1 & 2). At the point of extraction, temperature and relative humidity of the test gas are measured. The connection between main flow and exposure modules is made of chemically resistant Iso-Versinic tubes (Saint-Gobain Performance Plastics, Akron, USA). TYGON tubing (R 3603, Saint-Gobain Performance Plastics, Akron, USA) is used for most of the other connections in the system. The test gas enters the exposure modules through trumpet-shaped inlets in the lid. Inside the exposure modules, cells grown on membranes are supplied with basolateral culture medium while being exposed apically to the test gas. Cells exposed to humidified clean air in a VITROCELL 6/3 stainless steel exposure module (Clean air) are used as a control to the test gas. More technical details on the exposure modules can be found in a previous study [40]. The flow rate (0–20 mL/min) of the test gas across the cell monolayer is adjusted by using vacuum calibration valves connected to a second vacuum pump (Laboport N840FT.18, KNF Neuberger GmbH, Freiburg, Germany). Each position of the exposure modules has its own valve. Both exposure modules and the control module are connected to a heated water circuit (CC-106A, Peter Huber Kältemaschinenbau AG, Offenburg, Germany) to maintain a stable temperature inside the modules. Most of the devices, tubing and modules are housed in a climatic chamber. Temperature inside the climatic chamber is monitored (Pt100 type probes, testo 176 T2, testo SE & Co. KGaA, Lenzkirch, Germany), and heat is distributed by heaters and fans.

### 2.5 Adjustment of the ALI exposure system

Pre-experiments using humidified clean air as test gas were performed to validate the exposure system as well as to optimize the following experiment parameters to achieve high cell viability. In the first experiments, the heaters in the climatic chamber and the water circuit system were set to 37°C. The position of the heaters and fans in the climatic chamber and the flow direction of the water circuit in the hull of the exposure and control modules were in ‘standard settings’, which resulted in an uneven heat distribution inside the climatic chamber as well as a temperature gradient along the exposure and control modules, increasing the risk of losing humidity from the test gas due to condensation. In this setup, the relative humidity (rH) of the test gas entering the exposure modules was *calculated* to be 68–82% at 37°C after humidifying the dry feed gas (N_2_) in both dilutors (1:5 ratio) with humidified clean air which was *measured* to be at 85% rH. However, these conditions resulted in low cell viability in the pre-experiments. Therefore, we hypothesized that the rH of the test gas was lower than calculated, possibly due to lower temperature in the exposure system. To solve this problem, temperature of the heaters (40°C) and water circuit (38.5°C), as well as humidity of the feed gas (40 g/m^3^) were increased. Moreover, heaters and fans in the climatic chamber were repositioned for better heat distribution. Further, the flow of the water circuit for exposure and control modules was improved according to Leibrock et al. [41] in a way that the heated water entered the lids of the exposure modules first and the hulls second. This helps to prevent condensation, i.e., loss of water from the test gas at the inlets of the exposure modules. To verify that these adjustments contributed to create incubator-like ambient conditions during exposure, a RH/T Controller (Vitrocell Systems GmbH) was added to the exposure system. Inline RH/T sensors downstream of each dilutor and upstream of the exposure modules (red dots in Fig 2) were installed to monitor (Vitrocell Monitor, Version 1.05) temperature and rH of the test gas in proximity to the cells. Ultimately, a rH between 60–66% at 38°C was reached, which was enough to achieve high cell viability during experiments.

### 2.6 Cultivating lung cells on inserts at ALI conditions

**Fig 3:**
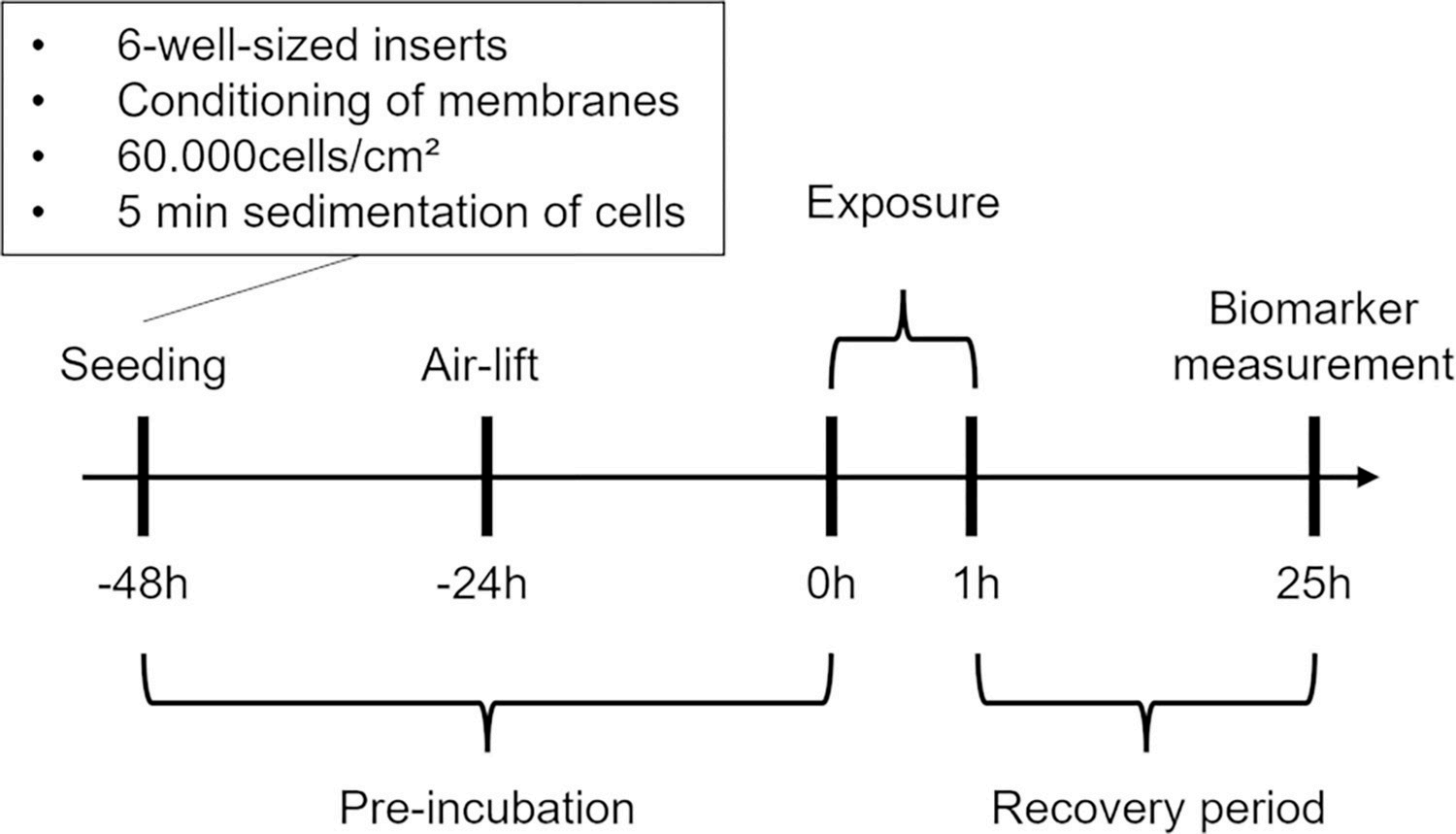
Timeline of air-liquid interface experiments.

Forty-eight hours before exposure, A549 cells were seeded on the apical side of 6-well-sized inserts (4.524 cm^2^ area, pore size 0.4 μm, transparent membrane, Greiner Bio-One, Frickenhausen, Germany) at a density of 60.000 cells/cm^2^ in 1 mL growth medium (Fig 3), according to Ruth et al. [42]. Before seeding, inserts without cells were placed into microplates (CytoOne, Starlab GmbH) filled with 1.5 mL growth medium in the basal compartment and were incubated for 30 min in the incubator to condition the membranes of the inserts. After seeding, the plates containing the inserts were not moved for 5 min to let the cells sediment onto the insert membrane, supporting a homogenous cell distribution across the growth surface of the insert membrane [43]. Then, A549 cells on inserts were pre-incubated at 37°C, 95% rH and 5% CO_2_. After 24 h pre-incubation, basal medium in microplates was aspirated and replaced with 1.5 mL ALI medium. ALI medium consisted of DMEM without phenol red (Gibco, 11880036) with 3% heat inactivated FBS, 25 mM HEPES (Gibco, 15630056), 4 mM GlutaMAX (Gibco, 35050038) and 100 U/mL penicillin/streptomycin (Gibco, 15140122). Medium on the apical side of the inserts was aspirated and the cell monolayer was rinsed with 1.5 mL PBS. Air-lifted inserts were pre-incubated for another 24 h until beginning of exposure.

### 2.7 Exposure of lung cells at the ALI

For information on calibrating the ALI exposure system, the reader is referred to S1 Text of the supporting information. As the temperature of the feed gas (N_2_) from the MGTB reached 37°C, the inlets of the exposure and control modules were carefully connected to the main flow and clean air of the exposure system, respectively. The whole system was stabilized for approx. 15 min before exposure started. Exposure started as the vacuum pump for the exposure and control modules was activated. At the same time, dosing of gaseous KEAA, O_2_ (20 vol%) and water (40 g/m^3^) from the MGTB started and A549 cells were exposed at the ALI to 50–2000 ppm C_3.7_ KEAA blend for 1 h at a flow rate of 20 mL/min. Humidified clean air (37°C, 85% rH) was used as a negative control (clean air control). Additionally, air-lifted cells in a microplate containing 1.5 mL basal ALI medium were incubated in the incubator to account for unspecific effects on viability of cells while being handled outside the incubator (incubator control). In separate experiments, NO_2_ (10 ppm) spiked humidified clean air was used as test gas to elicit a significant cytotoxic response from A549 cells at ALI conditions (positive control). Experiments were performed twice (n = 2) in four technical replicates for each KEAA blend concentration and NO_2_ or in triplicates for clean air and incubator controls, respectively. After exposure, the dosing and gas flow were shut down by closing the bypass from the MGTB and deactivating the vacuum pumps. Then, exposure and control modules were transported back to the sterile bench. Laminar airflow was briefly turned off again to prevent damage to the cells while transferring inserts from the modules and incubator control plate to new 6-well microplates containing 1.5 mL fresh basal ALI medium. Then, cells were post-incubated under ALI conditions for 24 h in the incubator.

### 2.8 Metabolic activity and lactate dehydrogenase release assay

After a 24 h recovery period in the incubator at ALI (Fig 3) metabolic activity and lactate dehydrogenase (LDH) release, as a marker for cell viability and necrosis of the exposed cells were assessed according to the manufacturer’s instructions. Briefly, an 1X alamarBlue HS Cell Viability Reagent (Life Technologies Corporation, Eugene, USA) solution was prepared in ALI medium. Inserts were rinsed with 1.5 mL PBS and 1 mL reagent solution was added apically to the cells. Then, inserts were transferred to new 6-well microplates without basal medium and incubated for 1 h in the incubator. After incubation, the supernatant reagent solution was homogenized and 100 μL supernatant per insert were transferred to a 96-well plate (CytoOne, Starlab GmbH) in duplicates. Fluorescence of supernatants was measured at 555/596 nm excitation/emission wavelength (Cytation 5 Multi-Mode Microplate Reader, Agilent, Waldbronn, Germany). 1X alamarBlue reagent solution in ALI medium was used as blank.

For investigating cytotoxicity of A549 cells, LDH release into the basolateral medium was measured using CyQUANT LDH Cytotoxicity Assay Kit (Life Technologies Corporation) according to manufacturer’s instructions. Studies showed that LDH content in the basolateral medium was higher compared to apical washes of A549 cell layers [37, 44, 45]. After the recovery period, 50 μL of homogenized basolateral medium per insert were transferred to a 96-well plate in duplicates and 50 μL of LDH assay reagent mixture were added. After 30 min incubation at room temperature in the dark, absorbance of samples was measured at 490 nm with a reference wavelength of 680 nm (Cytation 5). As an assay positive control, unexposed cells incubated at ALI conditions were lysed apically with 1.5 mL 1% Triton X-100 (TX1) 1 h before end of recovery period. After 1 h, supernatant medium was tested for LDH release as described above. ALI medium was used as blank.

### 2.9 Statistical analysis

Raw fluorescence and absorbance data were blanked, normalized, and expressed as percentage metabolic activity of clean air control or percentage of released LDH of assay positive control (TX1), respectively. To detect significant differences between controls and treatments, ToxRat Professional (Version 3.3.0, ToxRat Solutions GmbH, Alsdorf, Germany) was used. Depending on normality (Shapiro-Wilk test) and variance homogeneity (Levene test) of data, multiple comparisons of treatments against the clean air control (multiple sequentially-rejective Welsh-t-test after Bonferroni-Holm) or two sample comparisons between controls (Welch t-test or Kolmogorov-Smirnov test) was performed. Statistical significance was indicated as *p<0.05, **p<0.01, ***p<0.001 and ****p<0.0001. Figures were created with GraphPad Prism (Version 6.07, GraphPad Software, Inc.).

## 3. Results

### 3.1 Physicochemical analysis of feed gas and test gas

**Fig 4:**
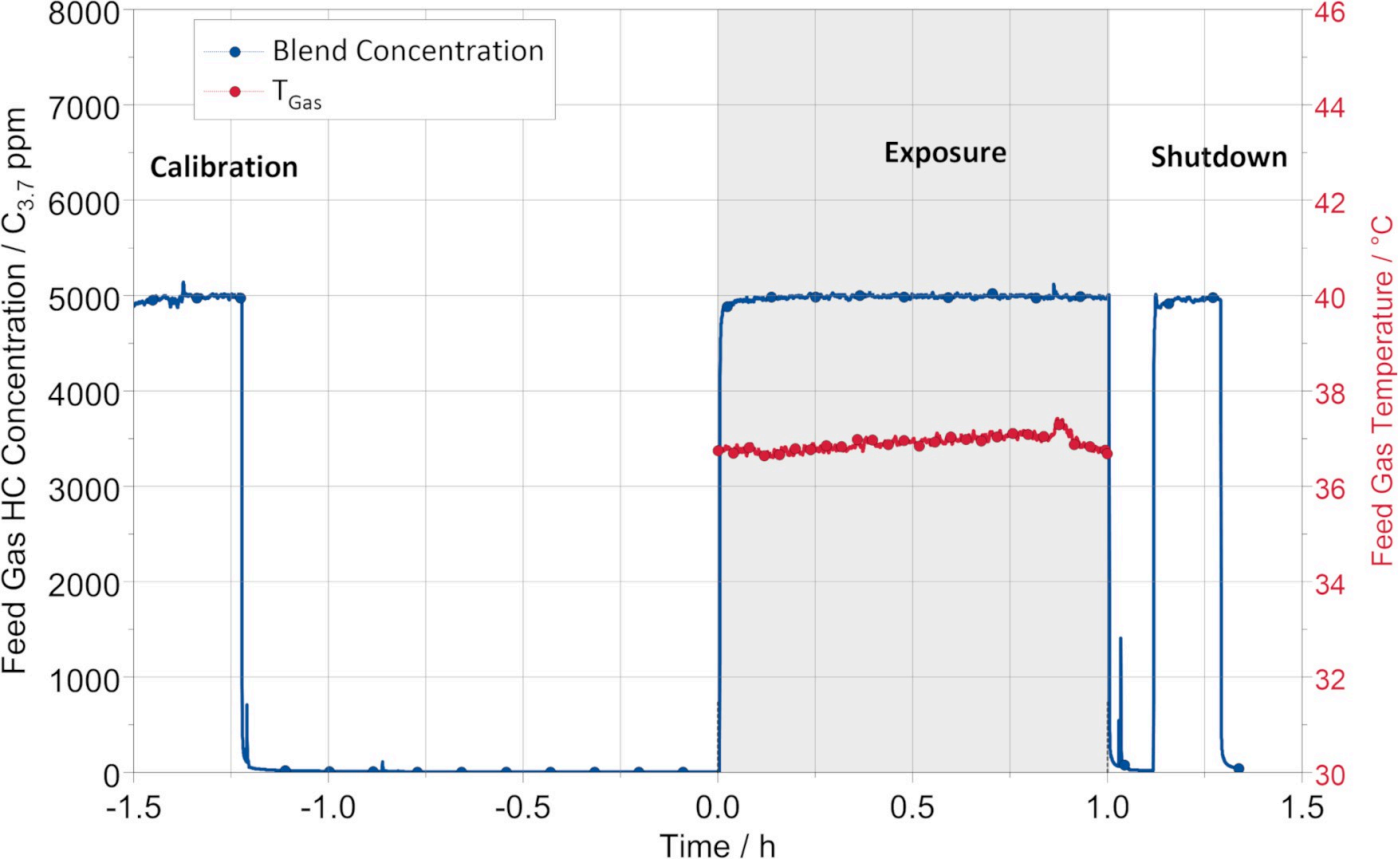
Representative feed gas concentration curve during the exposure procedure. The blue curve represents the blend concentration (KEAA) normalized to C_3.7_ in the feed gas before dilution in the exposure system. The red curve represents the feed gas temperature during the exposure phase, which is indicated by the gray background. Before (calibration) and after exposure (shutdown), the concentration level was additionally checked.

In Fig 4, a representative total hydrocarbon (HC) concentration (normalized to C_3.7_) of the dosed KEAA blend in the feed gas is shown. Concentration (5000 C_3.7_ ppm) and temperature (37°C) of the feed gas remained stable during exposure. Before (calibration) and after exposure (shutdown), additional checks confirmed stable HC dosing.

**Fig 5:**
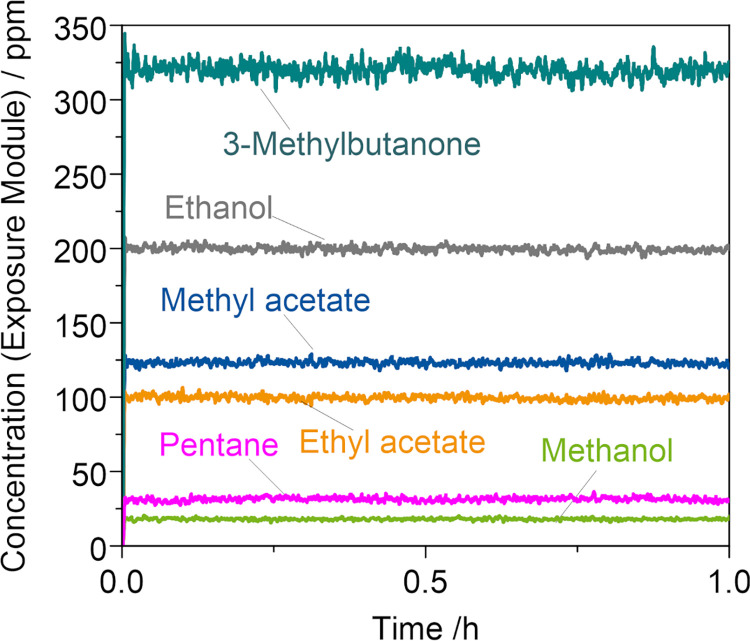
Representative calculated concentrations of single KEAA constituents after dilution of the feed gas during exposure.

In Fig 5, representative single concentrations of KEAA blend constituents in the test gas are shown for a total HC concentration of 1000 C_3.7_ ppm (1:5 dilution of feed gas containing 5000 C_3.7_ ppm KEAA). Calculated concentrations in the exposure module were stable during exposure and as follows: 3-methylbutanone (317.8 ppm), ethanol (198.3 ppm), methyl acetate (122.6 ppm), ethyl acetate (98.4 ppm), pentane (31.4 ppm) and methanol (17.7 ppm).

**Fig 6:**
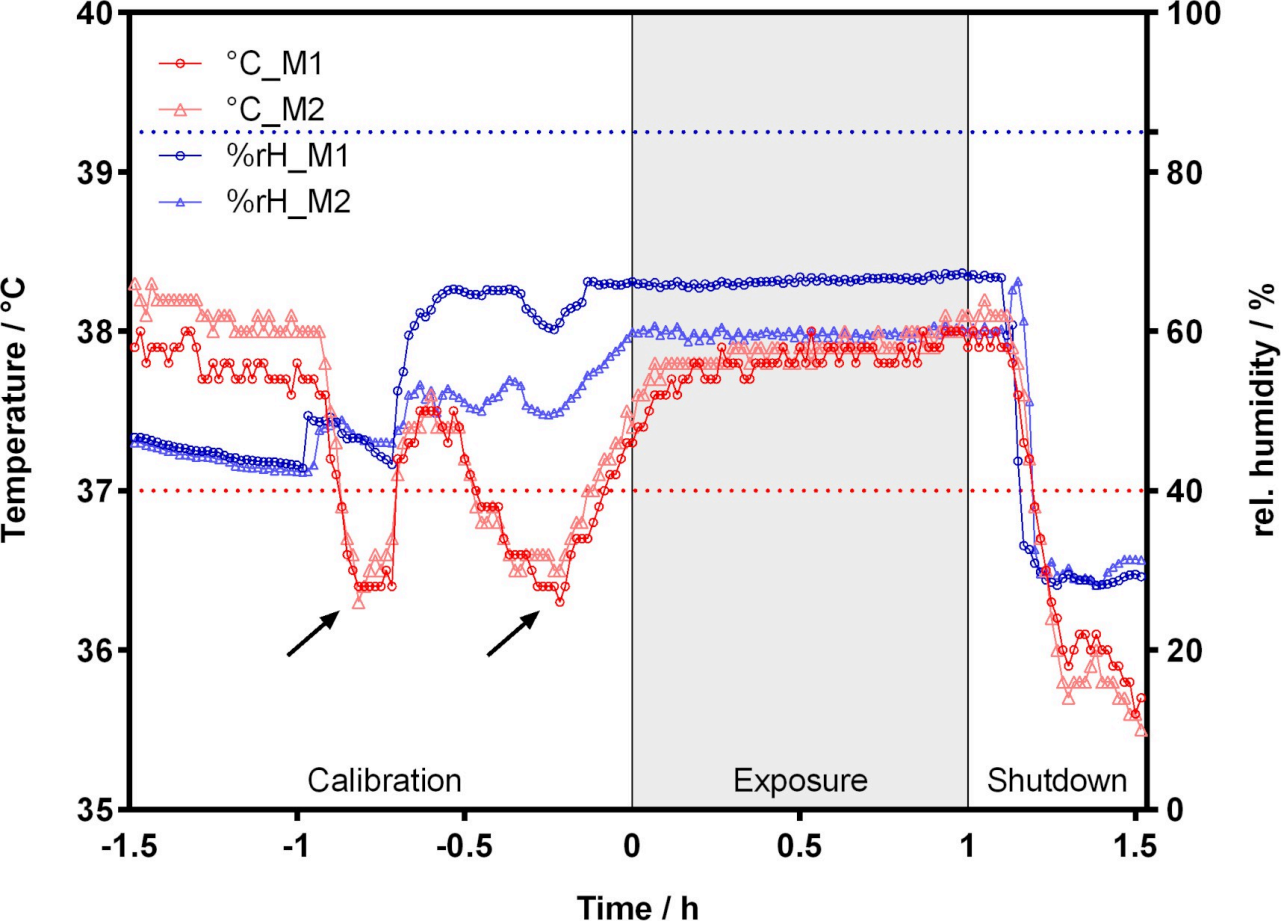
Temperature and relative humidity of the test gas during experiments. The temperature (red) and relative humidity (blue) of the test gas upstream of exposure module 1 (M1) and exposure module 2 (M2) were measured. The dotted horizontal lines indicate 37°C (red) and 85% rH (blue). Arrows mark the opening of the climatic chamber to insert exposure and control modules. Each experiment was divided into three phases: calibration, exposure, and shutdown.

Temperature and relative humidity (rH) of the test gas upstream of the two exposure modules were monitored during experiments (Fig 6). Before exposure (calibration), temperature and rH fluctuated due to opening of the climatic chamber to connect the exposure and control modules. During exposure, mean temperature was 37.8 ± 0.1°C and 37.9 ± 0.1°C for module 1 (M1) and module 2 (M2), respectively. Relative humidity was 66.3 ± 0.5% and 59.7 ± 0.5% for M1 and M2, respectively. After exposure (shutdown), temperature and rH dropped.

### 3.2 Adjustment of the ALI exposure system

**Fig 7:**
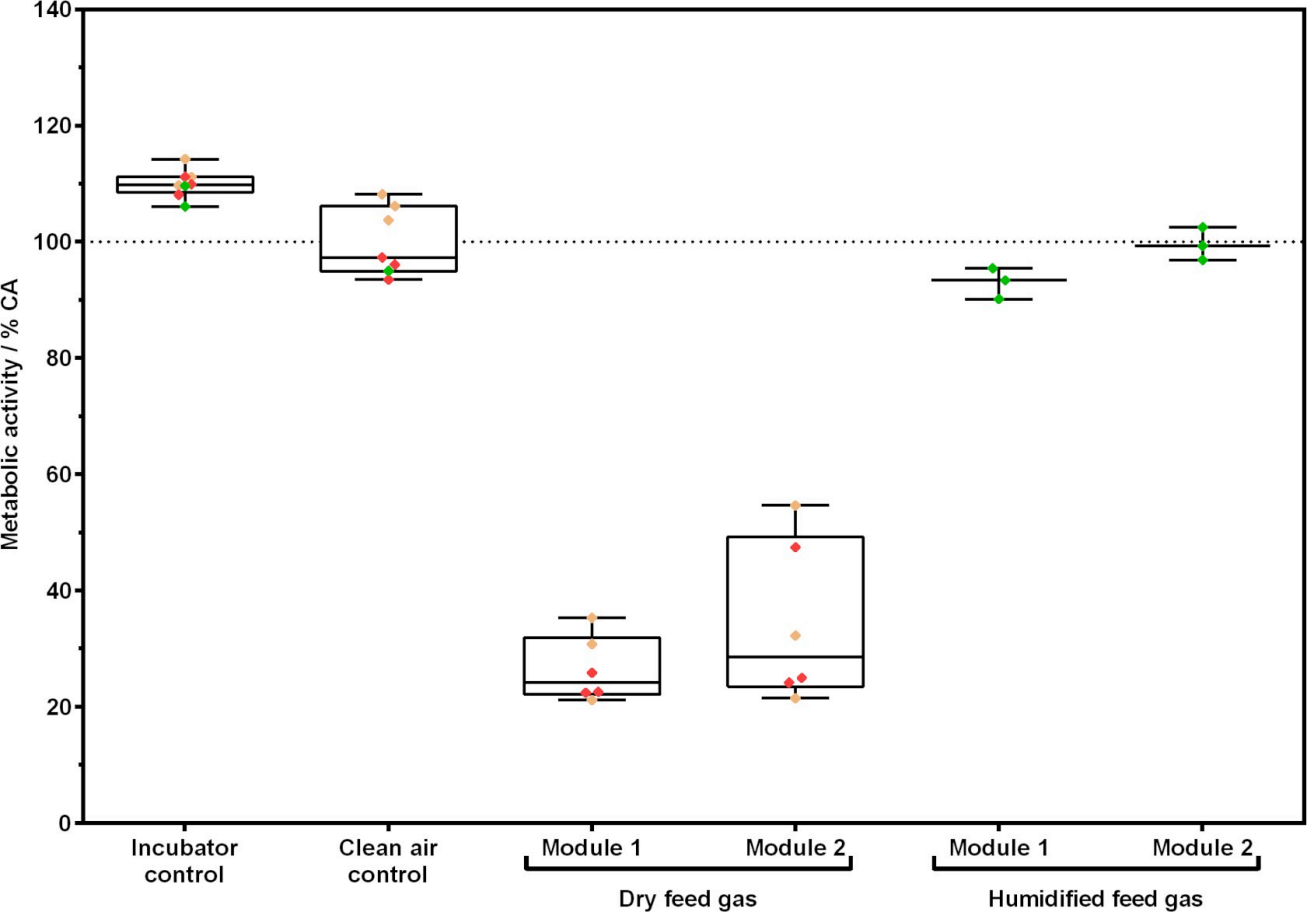
Metabolic activity of A549 cells after exposure to dry or humidified feed gas. Cells at ALI conditions were exposed to dry or humidified clean air for 1 h at a flow rate of 20 mL/min. After a 24 h recovery period at ALI conditions in the incubator, metabolic activity was investigated. Data was normalized to clean air control. Incubator control shows unexposed cells. Colored diamonds show technical replicates from independent experiments where one color refers to a set of two modules and controls.

Fig 7 shows the metabolic activity of A549 cells after exposure to dry or humidified feed gas. Metabolic activity was normalized to the clean air control. Cells exposed to dry feed gas in both exposure modules (Module 1 & 2) showed lower metabolic activity (26.4 ± 5.6% & 34.2 ± 13.7%) compared to cells exposed to humidified feed gas (93 ± 2.7% & 99.6 ± 2.8%).

Table 2 shows parameters measured before (standard) and after the ALI exposure system was adjusted (adjusted). Using adjusted settings, the temperature inside the climatic chamber and the exposure modules was higher compared to the standard settings. The mean cell viability in the clean air control (CA) was higher with a lower standard deviation for adjusted settings compared to standard settings (89.9 ± 8.6% and 66.7 ± 33.8%).

**Table 2:** Comparison of parameters for standard and adjusted settings of the ALI exposure system.

Parameters	Standard	Adjusted
Temperature inside climatic chamber	35.7 ± 0.7 °C	36.8 ± 0.2 °C
Cell viability in CA compared to IC	66.7 ± 33.8%	89.9 ± 8.6%

values are mean ± SD

### 3.3 Metabolic activity

**Fig 8:**
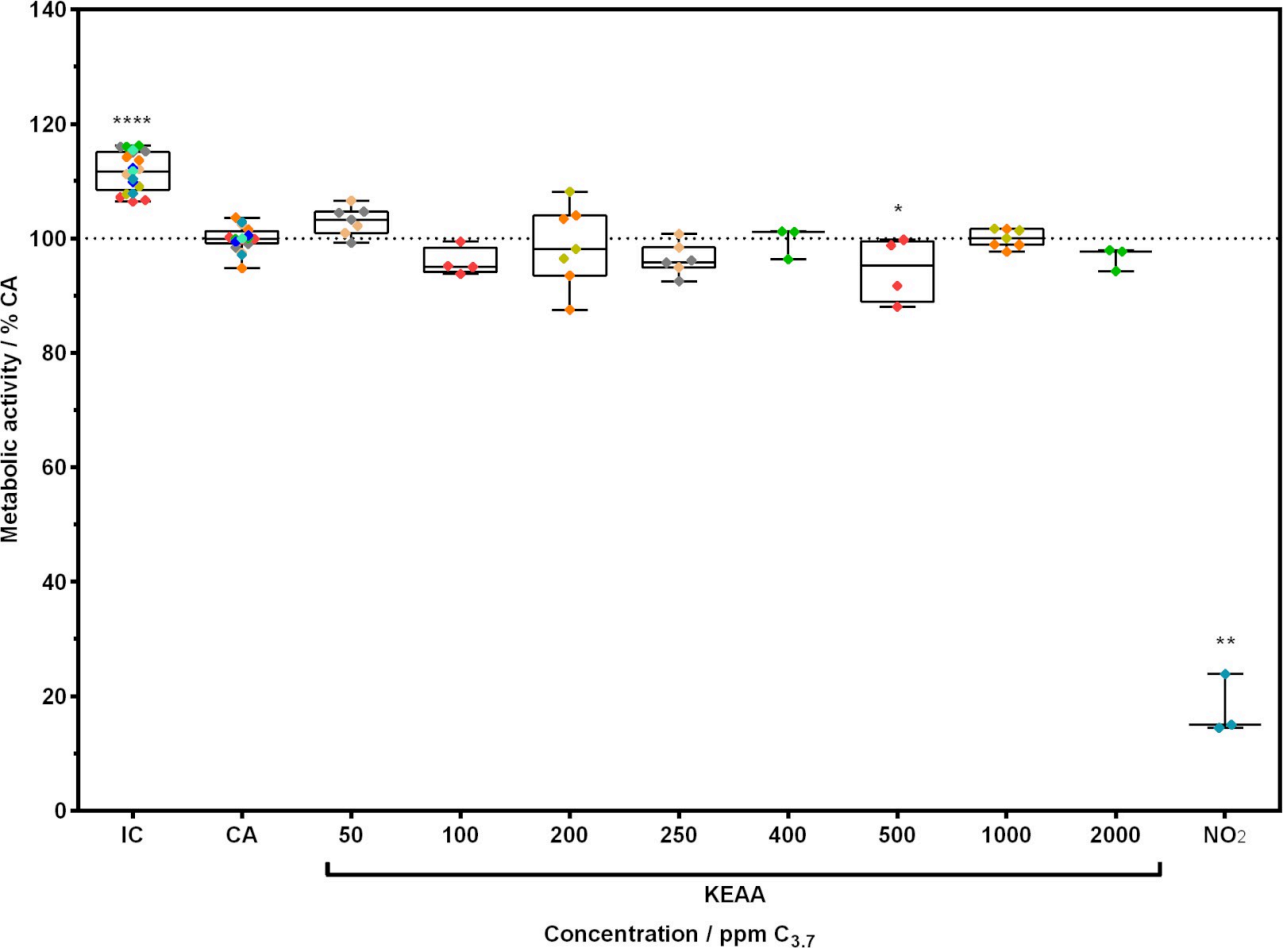
Metabolic activity of A549 cells after exposure to gaseous KEAA fuel blend. Cells at ALI conditions were exposed to humidified clean air spiked with different concentrations of KEAA or NO_2_ (10 ppm) for 1 h at a flow rate of 20 mL/min. After a 24 h recovery period at ALI conditions in the incubator, metabolic activity was investigated. Data was normalized and compared to clean air control (CA). Incubator control (IC) shows unexposed cells. Colored diamonds show technical replicates from independent experiments where one color refers to a set of two concentrations and controls.

As shown in Fig 8, metabolic activity in A549 cells exposed to 10 ppm NO_2_ was significantly lower compared to the clean air control (CA) (17.8 ± 5.3%, p = 0.0011). The incubator control (IC) showed significantly higher metabolic activity (111.7 ± 3.4%, p<0.0001) than the CA. Metabolic activity of cells treated with KEAA was significantly different from the CA for the 500 ppm C_3.7_ treatment only (94.6 ± 5.6%, p = 0.0478). However, no trend for concentration-dependent change in metabolic activity was observed.

### 3.4 Lactate dehydrogenase release

**Fig 9:**
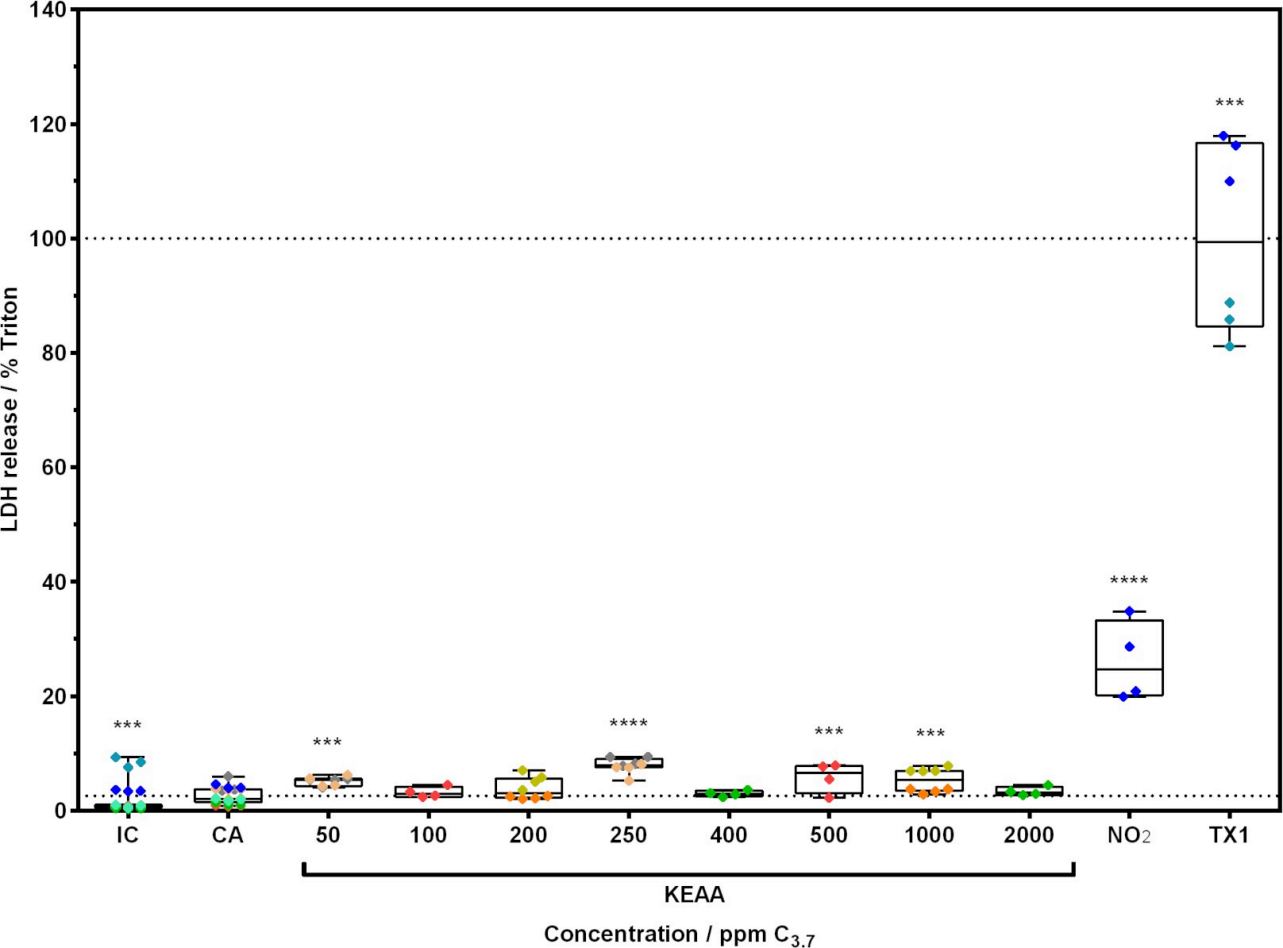
LDH release of A549 cells after exposure to gaseous KEAA fuel blend. Cells at ALI conditions were exposed to humidified clean air spiked with different concentrations of KEAA or NO_2_ (10 ppm) for 1 h at a flow rate of 20 mL/min. After a 24 h recovery period at ALI conditions in the incubator, LDH release was investigated. Data was normalized to assay positive control (TX1) and compared to clean air control (CA). Incubator control (IC) shows unexposed cells. Colored diamonds show technical replicates from independent experiments where one color refers to a set of two concentrations and controls. Lower horizontal line marks mean of CA (2.6%).

Cells exposed to 50, 250, 500 and 1000 ppm C_3.7_ KEAA showed significantly higher cytotoxicity as measured as LDH release (5.1 ± 0.8%, p = 0.0006; 7.9 ± 1.3%, p<0.0001; 5.8 ± 2.6%, p = 0.0011 and 5.3 ± 2%, p = 0.0002; respectively) compared to the CA (Fig 9). LDH release of the CA was 2.6 ± 1.4% of the assay positive control (TX1). Cells treated with TX1 and NO_2_ showed a significant increase in LDH release compared to the CA (100 ± 16.6%, p = 0.0001; and 26.1 ± 7%, p<0.0001; respectively). The IC showed significantly lower LDH release (1.9 ± 2.6%, p = 0.0001) than the CA.

## 4. Discussion

### 4.1 Temperature and humidity are key factors to maintain cell viability in ALI experiments

Although a significant difference in viability between incubator and clean air control was detected, this difference is comparable to what is reported in other studies [41, 46, 47]. Recently, studies conducting experiments with ALI exposure systems highlighted the need to check if the used exposure system provides physiologically relevant conditions during exposure [37, 41, 48]. Often the existing exposure system needs to be modified prior to performing experiments to obtain realistic and reliable data [39]. For example, keeping temperature and rH at stable incubator-like conditions, e.g., 37°C and 85% rH, is of high importance for survival of cells during ALI exposure. A study by Zavala et al. showed that low temperature and low rH of the vehicle control (clean air) resulted in cytotoxic effects and low cell viability in ALI experiments [37]. In our study we observed the same relationship between rH and metabolic activity of cells. Under these conditions, the potential effect of a test chemical could be masked by a high background toxicity due to poorly controlled exposure parameters. They also reported that only a small number of studies that conducted ALI experiments mention exposure parameters in detail. In our study, temperature and rH during experiments were at 38°C and 66% rH which were measured immediately upstream of the exposure modules. This point of measurement can only be a surrogate for the temperature and rH at insert level inside the exposure modules. However, the measured rH corresponds to 70% rH normalized to 37°C, which is still below the target of 85% rH reported in other studies [37, 48]. Despite this, according to Zavala et al. a rH below 85% could be practical since a lower rH at a certain temperature also means a lower dew point, which is the temperature at which water condenses in the air [37]. Further, a non-condensing atmosphere is desirable to protect downstream analysing devices [48]. The dew point for 38°C and 66% rH is approx. 30°C. In our experiments, we observed small condensation in the outlet tubes of the exposure modules although the tubes were at 36.8 ± 0.2°C, well above the calculated dew point. The condensation could have been excess humidity evaporating from the medium in the basal compartment of the exposure modules, as reported by Zavala et al. [37]. However, we observed no notably low volume of medium in the basal compartment of exposure modules after 1 h exposure. As mentioned, we installed a RH/T sensor upstream of each exposure module as a surrogate measurement for conditions at the insert-level inside the exposure modules. However, measurements upstream do not necessarily represent the conditions at the insert-level, where exposure of cells takes place. Guénette et al. observed a difference between measurements taken at the insert-level and downstream of the exposure module, suggesting not to rely on the surrogate measurements but to directly measure temperature and rH at the insert-level for better accuracy [48]. However, depending on the exposure system design, it could be difficult to place sensors at the insert-level for real-time monitoring of temperature and rH.

### 4.2 Delivery of test substances

Differences in concentration may arise from transport losses of test chemicals from chemical source to exposure module due to adsorption or reaction with pipes [33]. Since the pipes and tubes conducting the test chemicals in our experimental setup were made of stainless steel and inert tubing, we argue that adsorption or reaction was negligible. Guénette et al. established an ALI exposure system for testing of ozone with a feedback control loop that adjusts the ozone concentration at the source based on the downstream chemical analysis after the exposure module [48]. Kastner et al. used a downstream gas analyzer for formaldehyde and NO_2_ exposure [49]. When online monitoring of the test chemical cannot be achieved, different techniques for offline chemical analysis can be implemented. Bardet et al. analyzed the concentration of formaldehyde within the generation chamber using SPME on-fiber derivatization [50]. Persoz et al. put a passive sampler inside the exposure chamber and subsequently extracted the sampler after exposure for chemical analysis of formaldehyde [51]. A similar approach was chosen by Guénette et al. for delivery assessment of ozone [48]. A different approach is to perform deposition assays using reagents. Ritter et al. confirmed the delivery of NO_2_ onto the insert membrane using a reagent on the membrane that reacts with NO_2_ upon exposure which can be measured photometrically [52]. In our validation experiments, we performed a qualitative investigation of the delivery of NO_2_ onto the insert membranes by checking the inserts before and after exposure to 10 ppm NO_2_ for 1 h. After exposure, yellowish residuals distributed homogenously onto the insert membranes were observed which indicated deposition of NO_2_. Therefore, we reasoned that KEAA also was delivered to the exposure modules. Nevertheless, the effective concentration at cell level in the exposure module may have been different from the calculated concentration after dilution, which was based on the measurement of chemicals upstream of the exposure module.

### 4.3 Effects of gaseous KEAA fuel blend on lung cells

In our study, we aimed to create a relevant short-term exposure scenario. According to the GESTIS—International Limit Values for Chemical Agents [22] occupational exposure limits (OELs) of a given chemical must not be exceeded at the workplace. The OELs are used for workplace safety regulations and state average concentrations for both an 8 h work shift or a 15 min interval, in which the concentration can be higher than the 8 h value but must not occur more than 4 times during a work shift [53]. Since an 8 h exposure at ALI is practically difficult to accomplish, we designed a 1 h exposure scenario to equal 4 times a 15 min interval with high concentrations. This scenario represents, e.g., fuel-related tasks of vehicle mechanics like draining the fuel tank or changing the fuel pump of a car where high short-term airborne concentrations exceed OELs [21]. The DFG OELs for 15 min exposure to the KEAA constituents are 400 (3-methylbutanone), 800 (ethanol), 400 (methyl acetate), 400 (ethyl acetate), 2000 (n-pentane) and 200 ppm (methanol) [22]. The concentrations of the dosed single KEAA constituents for the highest tested KEAA concentration (2000 ppm C_3.7_) based on measurements and calculations were 635.6, 396.6, 245.2, 196.8, 31.4 and 35.4 ppm, respectively. In this case, only the concentration of 3-methylbutanone (635.6 ppm) exceeded the OEL by a factor of approx. 1.6. Looking at the cell responses, we observed a significantly different but uniform response in LDH release from cells treated with either low or high concentrations of KEAA compared to the clean air control. KEAA contains pentane which perturbates biological membranes, but only to a minor extent [54]. This may have led to the formation of pores in the cell membrane from which cytosolic biomarker, e.g., LDH can leach into the surrounding medium. However, since there was no concentration dependent increase in LDH release of exposed cells and mean LDH levels were not higher than 10% of the clean air control, we assume that the slight cell membrane damage was likely caused by unspecific background toxicity due to sub-optimal exposure parameters. This reasoning is supported by our data on metabolic activity of cells treated with KEAA. Here, only a slight but on the brink of being not statistically significant difference was found in metabolic activity of cells treated with a medium KEAA concentration compared to the clean air control. This leaves the question, whether a biological relevance of this significant difference can be attributed. In contrast, our results clearly show the significant cell damaging effect of NO_2_ which was also found in comparable studies [12, 49]. Therefore, our data support the OELs of the single KEAA constituents and moreover indicate no adverse effect to A549 cells when exposed to a mixture in the tested concentration range.

### 4.4 Limitations of the study

In general, ALI experiments are considered more sensitive and more physiologically relevant for investigating inhalation toxicity of gaseous compounds compared to experiments using cells submerged in culture medium [31, 55, 56]. However, there are biological as well as technical limitations of our study that may have impaired the sensitivity of the ALI exposure experiments.

Biological limitations include i) the use of the A549 human lung cell line. Despite its widespread use as *in vitro* lung model for ALI experiments [31, 42], there are drawbacks compared to primary cells. For example, A549 cells lack the ability to form tight junctions [35, 57]. Further, the metabolism and genotype of cell lines are altered because of immortalization or its origin from cancer tissue, which places cell lines far from *in vivo* conditions [58, 59]. While primary cells are fully differentiated and thus provide a considerably more realistic model of the human airway, cell lines need stimuli for differentiation to take place before *in vivo* characteristics are expressed [57]. Therefore, the data generated in our study needs to be verified using primary human lung cells in future experiments to account for discrepancies when extrapolating effects from cell lines to the *in vivo* situation. Another biological limitation could be ii) the timepoint of endpoint investigation was too far apart from exposure. For example, Friesen et al. observed a difference in responses of lung cells when endpoints were investigated 0 h, 4 h and 24 h after exposure to treated carbon fibers [60]. Technical limitations of our study include i) the exposure duration was short. Depending on the exposure system used, longer exposure durations of up to 4 h can be achieved [12, 41, 61]. Despite this, our aim was to establish a short-term acute exposure scenario, which is comparable to other studies in this field [49, 62, 63]. Since we observed a significant difference in cell viability between clean air control and incubator control, it is apparent that a slight background toxicity of the exposure system was present, most likely due to sub-optimal exposure parameters. As discussed in section 4.1, it is crucial to keep exposure parameters at incubator-like conditions for the whole exposure duration. Therefore, we need to improve our exposure system in this regard before performing longer exposure scenarios. A further technical limitation is ii) only qualitative data regarding deposition of any gaseous compound was obtained. This leaves the question whether the upstream measured concentrations of the investigated fuel blend in the feed gas were comparable with the concentrations at insert level. Therefore, it can not be ruled out that, for example, the toxicity of the fuel blend was underestimated in the case that only a fraction of the dosed fuel blend was present at insert level. To link the effects observed in lung cells to a fuel concentration, trapping of chemicals inside the exposure modules via adsorbents [51, 52] or downstream washing bottles prove a feasible way [64]. For future experiments, we plan to use washing bottles as sampling method for downstream chemical analysis.

## 5. Conclusion and recommendations

Before experiments with the bio-hybrid fuel were performed, validation experiments were conducted to ensure high viability of A549 cells when exposed to humidified clean air. In our first experiments, viability of A549 cells exposed to humidified clean air was much lower than the incubator control and a high variance between replicates and experiments was observed. We identified temperature and rH as key contributors to the observed variance in the experiments. Therefore, we modified our exposure system to stabilize and monitor temperature and rH of the test gas more accurately in proximity to the cells. As a result, cell viability increased and variance in the data decreased.

In this study, A549 cells were exposed to a dynamic air flow of a freshly generated test gas containing a gaseous Ketone-Ester-Alcohol-Alkane (KEAA) bio-hybrid fuel blend. The concentration of KEAA was monitored online upstream of the Vitrocell exposure system. Data showed that stable and reproducible concentrations between experiments were achieved. However, the concentration upstream may differ from the effective concentration inside the exposure modules where exposure of cells takes place. A549 human lung cells were exposed at the ALI to 50–2000 ppm C_3.7_ gaseous KEAA for 1 h at 20 mL/min, 38°C and 66% rH, which were measured immediately upstream of the exposure modules. After 24 h recovery, there was a significant difference in LDH release and metabolic activity between clean air control and treatments in the tested KEAA concentration range. However, no trend for concentration dependent change in LDH release or metabolic activity of cells was observed. This indicates that the significant differences observed might be artifacts due to a yet working but still improvable setup of the exposure device. For future experiments, focus on high relative humidity of the test gas and measurement of the effective concentration of chemicals by offline deposition or online downstream analysis is important to increase reliability of the data. This combination makes ALI experiments a suitable tool in early-stage bio-hybrid fuel research in the sense of green toxicology. Moreover, this data can help decision makers or industry to better regulate potential gaseous hazards emitted from the transport sector. Our findings apply only to the exposure scenario tested in this study including the discussed drawbacks on the reliability of the effective concentration and is difficult to extrapolate to the real world since the investigated monoculture of A549 cells do not represent the complex *in vivo* situation.

## Supporting information

S1 Text Materials and methods. (DOCX)
