# Supplementary material for: Air-liquid interface exposure of A549 human lung cells to characterize the hazard potential of a gaseous bio-hybrid fuel blend
Source: PLoS One. 2024 Jun 24;19(6):e0300772. doi: 10.1371/journal.pone.0300772 (PMC11195957; doi:10.1371/journal.pone.0300772)
Supplement: S1 Text — (DOCX) [file pone.0300772.s001.docx]

**S1 Text. Materials and Methods**

## Cultivation of lung cells

A549 cells were obtained from the German Collection of Microorganisms and Cell Cultures GmbH (DSMZ, ACC 107, Braunschweig, Germany) and cultured in growth medium (DMEM, Gibco 21885108, Fisher Scientific GmbH, Schwerte, Germany) supplemented with 9 % heat inactivated FBS (Gibco 10500064) at 37 °C, 95 % rH and 5 % CO_2_. Cell culture medium of subconfluent cells was aspirated, cells were rinsed with PBS without ions (Sigma-Aldrich Chemie GmbH, Taufkirchen, Germany) and detached from cell culture flasks (CytoOne, Starlab GmbH, Hamburg, Germany) using a 0.05/0.02 % Trypsin/EDTA solution (SAFC, Sigma-Aldrich). Trypsination was stopped with growth medium and cells were reseeded in fresh growth medium. For experiments, A549 cells at passages 7-20 were used.

## Calibrating the ALI exposure system for experiments

Before the start of exposure, the two clean (rinsed with 70% ethanol) exposure modules and the control module for clean air were connected to a water bath and pre-warmed to 37° C for approx. 2 h. Then, under sterile conditions 6.7 and 19.7 mL of pre-warmed ALI medium were dispensed per slot in the exposure modules and in the control module, respectively. The difference in volume between exposure modules and control module was due to different cup sizes per module slot. This difference only applied for the exposure phase. During the subsequent post-incubation, the media volume for all treatments was identical. Therefore, dilution effects can be ruled out. The air-lifted inserts with A549 cells were taken out of the incubator, placed into the module slots, and checked for air bubbles below the membranes. During this step, the laminar airflow of the sterile bench was briefly turned off until the inserts with cells were enclosed within the modules, avoiding A549 cells drying out. The distance between the trumpet-shaped inlets and inserts inside the modules was 2 mm. Then, the modules were put into the climatic chamber and were connected to the water circuit. Until connection to the main flow or clean air, respectively, the inlets of the modules remained sealed with rubber plugs. Approx. 2 h before the beginning of exposure, both the water bath for the humidifier and the water circuit for the exposure and control modules were set to 38.5 °C. The climatic chamber was heated overnight to 40 °C. The closed bypass transporting the feed gas from the MGTB to the exposure system (Fig 1) was connected to the main flow of the exposure system and was opened as the feed gas reached temperatures below 40 °C to prevent damage to the exposure system. At the same time, the main flow vacuum pump (5 L/min) and dosing of clean air were activated. Then, mass flow, pressure, temperature and rH of the main flow and clean air were calibrated using nitrogen as feed gas. The vacuum pump for the exposure and control modules was briefly activated to set the vacuum valves for the flow rate across the cells to 20 mL/min using a calibration meter (GFM 17, ANALYT-MTC Messtechnik GmbH, Müllheim, Germany). During calibration, which took approx. 1.5 h, the exposure and control modules remained disconnected from the main flow and clean air, respectively, leaving the cells undisturbed.

## Humidity of the test gas

The water evaporator was set to 40 g H_2_O/m³, which meets the target relative humidity of the test gas of 85% at 37° C. However, this target was not reached during experiments. We have two possible explanations for this: First, the distance between upstream water evaporator and downstream exposure system might have led to a temperature gradient along the (heated) gas lines, favoring condensation of water from the feed gas. In fact, when disconnecting the bypass of the model gas test bench from the exposure system after experiments, we observed water dripping out of the bypass. However, during shutdown phase after exposure, the temperature of the test gas drops below 20 °C (see Fig 6), which might have been the origin of the condensed water in the bypass. Second, there might have been a temperature gradient along the clean air gas line within the exposure system. This would mean that downstream of the relative humidity measuring point of the clean air (see Fig 2) water condensed from the clean air. Consequently, a clean air of lower relative humidity (<85%) would have been used to dilute the feed gas, decreasing the overall relative humidity of the diluted test gas measured downstream of the dilutors. For future experiments, the experimental setup needs to be revised to achieve the target relative humidity of 85%.

Table S1. List of abbreviations.

| A549 | Human lung cell line |
| --- | --- |
| ALI | Air-liquid interface |
| BTEX | Benzene, Toluene, Ethylbenzene, Xylol |
| CA | Clean air |
| DFG | German Research Foundation |
| DMEM | Dulbecco’s Modified Eagle Medium |
| FID | Flame-ionization detector |
| FSC | Fuel Science Center |
| FT-IR | Fourier-transform infrared |
| HC | Hydrocarbons |
| IC | Incubator control |
| KEAA | Ketone-Ester-Alcohol-Alkane |
| LDH | Lactate dehydrogenase |
| M1 & 2 | Module 1 & 2 |
| MFC | Mass flow controller |
| MGTB | Model gas test bench |
| OEL | Occupation exposure limit |
| PBS | Phosphate buffered saline |
| PMD | Paramagnetic detector |
| rH | Relative humidity |
| RH/T | Relative humidity/Temperature |
| SPME | Solid phase microextraction |
| TX1 | 1% Triton X-100 |
